# Supplementary material for: Texting Brief Podcasts to Deliver Faculty Development to Community-Based Preceptors in Longitudinal Integrated Clerkships
Source: MedEdPORTAL. 2018 Sep 21;14:10755. doi: 10.15766/mep_2374-8265.10755 (PMC6342434; doi:10.15766/mep_2374-8265.10755)
Supplement: Supplementary file 1 — A. Podcast 1 Encouraging Continuity.mp3 B. Podcast 2 Bedside Teaching.mp3 C. Podcast 3 Encouraging Student Ownership of Patients.mp3 D. Podcast 4 Communicating and Managing Patient Results During Off-Clinic Days.mp3 E. Podcast 5 Choosing the Right Patients for Continuity.mp3 F. Podcasts 1-5.pdf G. Pre- and Postexperience Surveys.pdf [file mep-14-10755-s001.zip › F._Podcasts_1-5.pdf]

Podcasts written form

Encouraging continuity

Written by Dr. Joshua Bernstein, Director of the Internal Medicine Clerkship and Didactics at the Longitudinal Integrated Clerkship at the University of North Carolina, Asheville campus.

One of the greatest joys of practicing medicine is the relationships physicians build with their patients. In traditional medical school third year programs, students often are assigned to a clinic for a set block of time. Students will generally see patients once or perhaps twice during the rotation. This leaves little opportunity to establish meaningful relationships with patients. In longitudinal programs, students have the opportunity to see patients back in follow up on a regular basis and have an opportunity to build meaningful relationships over time. These relationships allow students to recognize the patient, not as a disease state, but as a person with a family, a job, hobbies, etc. Longitudinal programs improve humanism because of the relationships students build with their patients. Students in LICs also have the unique opportunity to follow disease processes over time. However, it can take some effort to foster this connection with particular patients. Preceptors have to encourage this student/patient continuity. There are really two main categories of continuity in longitudinal programs. The first is in office continuity and the second is out of the office continuity.

What are some strategies for encouraging in office continuity? First of all, choosing the right patient to follow is extremely important. This was covered in another podcast. How can we make sure the student has the opportunity to see the patient again in our clinics? Students will generally have a specific time and day of the week that they are assigned to be in clinic. Students' patients should be encouraged to make appointments on that specific day. Often the patients themselves can be the most helpful route to making this a success. As the visit is concluded, patients are told to please follow up in 3 months, on a Thursday morning. On that day our student will be there and will have the opportunity to see their patient in follow up.

According to Dr. Bernstein, office staff are also important in encouraging continuity. Staff should be educated on the goals of an LIC program, how scheduling can work and they should understand the importance of students seeing patients on a recurrent basis. If a nurse, MA, or secretary recognizes that a student's patient is calling, they could preferentially choose the student day for the appointment. Some EMR's will allow certain patients to be labeled as special or "student' patients. Depending on the system used these patients will then show up on the schedule in certain colors or fonts and then can be recognized as student patients. At the beginning of the week, the schedule can be reviewed and if a student patient is scheduled on a day other than when the student is there, then they can be called and asked if they can reschedule for the student's assigned day.

This continuity is invaluable in education. Students can see how earlier interventions or decisions have impacted health outcomes and how patients are progressing. Additionally, it helps form the student/patient relationship that is so important in providing care.

As Dr. Bernstein explains, patients often are seen in many other health care settings throughout the year. Each of these contacts is a potential learning experience for the student. Some examples may be specialist appointments, hospitalizations, or even at an ancillary service such as physical therapy. Students often get the experience of seeing patients referred to physical therapy, but rarely actually see what a physical therapist does during an hour session with a patient. During specialist appointments students can actually act as the patient advocate. For example, the student may help explain to the cardiologist that his or her preceptor was concerned about the possibility of bradycardia because the patient had been complaining of dizziness. Surgeries, ER visits, and radiology studies are all great examples of other continuity experiences that can be extremely rewarding for student learning.

How can we help encourage these continuity experiences? It takes effort on the part of the student and preceptor. At the start of the year, clear expectations should be set so that students understand that longitudinal experiences with their patients are expected. If students have unscheduled afternoons then these should be used for continuity experiences when possible. Often, just having the preceptor mention continuity can be helpful. A preceptor may say to a student "it would be great if you could go with Mr. Jones to her Oncology visit". Referral specialists should be enlisted as another source of help in this. At the start of the year, office staff should be notified of any student unscheduled days or sessions, and should be instructed to attempt to schedule, patients with tests or appointments on days when the student may have the opportunity to go. Lastly, the patients themselves may be the most useful source of making continuity happen. The student may say to the patient: "Mr. Jones, I really want to come with you during your consultation with the surgeon so that I can hear what he has to say about your hernia. Please call me on my cell phone when you find out about your appointment and I will try and meet you there." Preceptors should hold students accountable and ask students to report back on continuity experiences outside of the office.

Lastly, hospital admissions are a great source for continuity. Many longitudinal programs are largely outpatient based and having students gain extra time in the hospital can be beneficial. If a student's patient is admitted, the student should be notified, and expected to round on the patient and report back to the preceptor on how the patient is doing. Students can learn about not only the disease process, but also how the disease is effecting the patient directly. Some preceptors choose to notify students anytime one of their patients are admitted even if they are not yet established with the student. The student may meet that patient for the first time in the hospital, but then upon discharge the patient can follow up in the clinic and the student can gain experience in transitions as well as then establish a relationship with the patient.

Encouraging continuity takes effort, and can sometimes feel uncomfortable for students. However, the value in forming relationships with patients and seeing how their medical problems are approached in different healthcare settings is an invaluable experience and should be encouraged whenever possible. Taking the time to make this a priority will ultimately help students succeed in longitudinal programs.

Bedside Teaching

Written by Dr. Sandra Whitlock, Associate Program Director of the Longitudinal Integrated Clerkship at the University of North Carolina School of Medicine, Asheville Campus.

Bedside case presentations can be an extremely effective tool for teaching medical students in the office setting. Despite once being a cornerstone of medical education, bedside teaching has been on the decline, possibly due to unfounded concerns about patient receptivity, time constraints, and learner discomfort. Today's podcast will address these concerns and focus on the benefits of bedside case presentations in the ambulatory setting.

First, let's listen to an example of a bedside presentation in a primary care office setting. Please pay particular attention to how the preceptor addresses the patient's comfort and engages him in the teaching encounter.

Doctor: Hi, Mr. Smith. How are you today?

Patient: Just OK today, Dr. Thomas. I haven't been feeling that well.

Doctor: I would like to hear from our student Sarah about how things have been going with you. I am going to listen carefully, but not interrupt, while she is sharing your story. Please feel free to chime in if you have anything to add or clarify. When Sarah finishes, we will talk about the plan in more depth. Also, we will do our best to avoid speaking in medical lingo because we don't want to exclude you from our conversation, so please let us know if you don't understand something.

Patient: That sounds good.

Student: Mr. Smith is our 61 year old patient with a history of hypertension and high cholesterol who is coming in to the office today with a complaint of lower back pain. He states that he was doing yardwork when he stepped in a hole and fell backwards, landing hard on his back. This happened about 5 days ago. He has had a lot of pain with movement. He has taken Tylenol and ibuprofen without much benefit.

Patient: Actually, I just took ibuprofen that one time, but it upset my stomach. Tylenol hasn't been very helpful.

Student: Right, OK. He hasn't noticed the pain radiating…I mean, spreading, anywhere, such as down his legs. He hasn't noticed any numbness or tingling. On physical exam, he did not have any tenderness to his spine or muscles. He did not have any pain down his legs with straight leg raise. He had normal strength, sensation, and reflexes in his

legs. So, I think this is probably musculoskeletal back pain, but I wasn't sure if we should get an X-Ray because of the fall.
Patient: I was wondering about that, too.
Preceptor: OK, that was an excellent presentation. Mr. Smith, is there anything else you would add?
Patient: That pretty much sums it up, but I'm worried that I might have really messed up my back. It's been pretty frustrating not to be able to do anything.
Preceptor: I understand. It sounds like this has been really hard for you. I know that you're an active person who doesn't like to sit still. First of all, I am hopeful that we can help with your pain, so I want to reassure you about that. But first, I'd just like to ask just a few more questions to make sure that there aren't any other causes of your pain that we should be considering…..
(Fades out)

As illustrated in this scenario, patients can be made to feel like active participants in their own care and the teaching process by having these case presentations in the examination room. Despite concerns about patient acceptance, bedside case presentations have actually been shown to increase, rather than decrease, patient satisfaction. Patients do not wait as long in the examination room by themselves and instead have more face-to-face time with the attending and student. Patients typically report a high level of comfort with listening to their case discussed in the room. After participating in a medical student presentation, patients are also more likely to feel that medical students contribute to their satisfaction with the visit. The key is to be able to set up the encounter in a way that includes patients in the conversation, rather than talking over them. In this example encounter, the preceptor introduced the concept of the bedside presentation and gave permission to the patient to interrupt if he needed to clarify information. The preceptor also made a statement about trying to avoid medical jargon, which simultaneously reassures the patient and reminds the learner and preceptor to talk in a way that the patient will understand. Prior to seeing the patient, it is also crucial that the preceptor ask the student about potentially sensitive subjects or serious diagnoses that may be considered, since students may still be learning the skills to handle these issues delicately.

Most preceptors find that bedside case presentations do not take more time, while others feel that they can actually *improve* efficiency. In a traditional presentation model, a student presents the patient's case to the preceptor outside of the examination room, either in a conference room or hallway. The student and preceptor then go into the room to confirm the history, complete the physical exam, and discuss the care plan, thereby adding an extra step in the process of teaching. The care plan may need to be altered if the preceptor obtains additional or conflicting history. As you heard in the sample interaction, bedside teaching eliminates this extra step and allows the patient to immediately verify or correct aspects of the medical history, allowing the student, preceptor, and patient to develop the appropriate care plan with fewer potential missteps. Experienced

preceptors may also choose to start their clinical documentation while listening to the presentation.

Students and preceptors will develop a comfort and perhaps even a preference for bedside presentations once they have more experience with this model. These interactions provide valuable opportunities for students to learn more about physical diagnosis, patient-physician communication, and humanism in medicine. It is also more likely that students will receive valuable feedback from their preceptors about their interactions and rapport with patients since these interactions are observed. With more time in the exam room together, preceptors can have more opportunity to model humanism. In this case, perhaps the student would notice the way the preceptor reassuringly put her hand on the patient's shoulder when discussing his level of distress.

In summary, bedside case presentations can be an effective and time efficient means of teaching students while caring for your patients. Studies have shown that when compared with standard presentations, bedside presentations improve satisfaction of patients, students, and preceptors. Patients will gain value from being active and engaged contributors in their own medical care and the educational process. Over time, students and preceptors can develop increasing comfort with this model, which may ultimately lead to more enjoyment and quality in overall office based teaching.

Alguire PC, DeWitt DE, Pinsky LE, Ferenchick GS, editors. Teaching in Your Office: A Guide to Instructing Medical Students and Residents, 2nd edition. Philadelphia: ACP Press; c2008. 178 p. (Ende J, editor. Teaching Medicine Series).

Rogers HD, Carline JD, Paauw DS. Examination room presentations in general internal medicine clinic: patients' and students' perceptions. Acad Med 2003; 78:945-949.

Encouraging Student Ownership of Patients

"Let me tell you about my patient": The **FACT** in medical student ownership in LICs
Written by Dr. Joanna Drowos, Associate Chair of the Department of Integrated Medical Science and Director of the Community and Preventive Medicine Clerkship at the Charles E. Schmidt College of Medicine at Florida Atlantic University

Among medical students participating in clinical training, "**ownership**" of patient care is important to develop and demonstrate. Ownership is a broad term, encompassing various aspects of professionalism, patient care and patient safety. It is also important to remember that medical students are expected to receive graded responsibility for patient care as they progress through their training. In figuring out how to best support and encourage patient ownership by students, it is

important to first define the expected behaviors that constitute ownership, and remember that as preceptors we need to both model these behaviors, as well as facilitate an office environment that provides opportunities for engagement in these kinds of behaviors.

As Dr. Drowos points out, a good mnemonic for remembering these principles is **FACT**, which stands for **FOLLOW-THROUGH**, **AUTONOMY** and **ADVOCACY**, **COMMUNICATION** and **COMMITMENT**, and **TEAMWORK**. These together with **KNOWLEDGE**, are the important behaviors that demonstrate your "**ownership**" over patient care. Listen for these key words throughout the podcast.

So what does it look like when a student takes ownership of their patient? We expect students to show **COMMITTMENT** to their patient – that they will put their professional responsibilities first, be actively engaged and participate in the care of the patient. They will also be invested in the outcome of what happens to their patient. We expect that they will go the extra mile for a patient, and along the same lines, practice **ADOVACY** on behalf of their patient. This includes being vocal or assertive about what they feel is in a patient's best interest and even raising questions to the team as appropriate. **AUTONOMY** is important – we want to see our students taking increasing independence for patients, however they also need self-awareness of their own limitations and to understand when consultation is needed. We expect students to think critically and stand behind their decision-making. This requires appropriate **KNOWLEDGE**, both in reading and learning about their patients and in their general clinical knowledge base.

COMMUNICATION skills encompass effectively relaying information to patients, their families, other providers and health care team members, and are particularly important when a transition in care is occurring. Collaboration and **TEAMWORK** are essential to demonstrate, both in an awareness of one's role within the team, and through sharing responsibility with other team members appropriate for the care of the patient. Lastly, **FOLLOW-THROUGH** in carrying out a treatment plan, taking care of details and making sure that any issues do not fall through the cracks, is an important part of taking ownership.

So how can we best help our medical students behave in ways that demonstrate **FOLLOW-THROUGH**, **AUTONOMY** and **ADVOCACY**, **COMMUNICATION** and **COMMITMENT**, and **TEAMWORK**? These behaviors, along with **KNOWLEDGE** are the **FACT**s that can help our students take **OWNERSHIP** of their patients. The most important way, is by demonstrating these behaviors ourselves. We can show our own knowledge by "thinking out loud" and sharing our clinical reasoning and theories with students. When we take the time to personally call a patient or another physician to answer questions or discuss a case, we should allow the student to not only witness this, but to participate in the conversation. Even if it is not us directly, but someone in our office who is taking the time to provide justification and help a patient with a needed authorization from their insurance, we

should involve the student in this as well. If we are waiting for a result and need to move on to another patient, we need to model the way that we close these loops-going back to check and making sure that no information is missed. Ultimately the goal is to have the students be able to perform these activities independently, without prompting and to hold them accountable for what is best for the patient. One of the best ways to promote this, is to select a smaller cohort of patients that the student is responsible for. This really gives a student the opportunity to get to know and to engage with a smaller group of patients, rather than expecting that they will know or engage with all patients. Choosing appropriate patients for continuity is covered in another podcast. Having students perform functions such as these may end up adding value for patients, and for our offices.

Dr. Drowos suggests that examples of opportunities for students to be engaged in your office include having them help with pre-visit planning, allowing them to put in orders for preventive services where appropriate, reviewing orders for medication refills, determining what labs are needed, and calling patients in advance of visits to discuss any pertinent issues and plan an agenda for the visit. Students can also meet patients in the hospital prior to discharge to assist in managing their transition, or attend a specialist visit with them to provide a link back to their referring provider. Students can develop and give patient education talks on common clinical problems, such as upper respiratory infections or constipation. Students can also review social histories and should participate in huddles in order to understand issues that can impact patient care. Between clinic sessions, students should be expected to call and check on patients when appropriate, attend specialist visits with them, or even call with lab results once the student and preceptor agree that the student is ready for this step and they have been trained on appropriate documentation.

Patient engagement takes work and effort. It should be the responsibility of the preceptor to encourage this ownership and to hold students accountable. Perhaps before clinic or during the week preceptors could ask the student directly…"so how is Ms. Jones doing with the antibiotic we prescribed" or did you get a chance to call Mr. Smith after his recent hospital d/c to see how he is doing"? This not only models follow-through, but provides unique learning opportunities.

Students do not want to be passive observers. In order to encourage our students to take ownership of their patients, we need to clarify our expectations around demonstrating **FOLLOW-THROUGH**, **AUTONOMY** and **ADVOCACY**, **COMMUNICATION** and **COMMITMENT**, and **TEAMWORK** in addition to **KNOWLEDGE**, that can help our students take **OWNERSHIP** of their patients. We also need to make sure to model these types of behavior ourselves, and to include students in the types of interactions where we demonstrate these behaviors. We also need to work with our office staff to create an environment where students will have opportunities to witness, learn about and ultimately perform these behaviors that will allow them to demonstrate ownership of their patients.

Reference:

McLaren K, Lord J, Murray SB, et al. Ownership of patient care: a behavioral definition and stepwise approach to diagnosing problems in trainees. Perspectives on Medical Education. 2013;2(2):72-86. doi:10.1007/s40037-013-0058-z.

Communicating and Managing Patient Results During Off-Clinic Days

Written by Dr. Tali Ziv, Associate Program Director of Kaiser-UCSF Longitudinal Integrate Clerkship, and Dr. Lindsay Mazotti, Assistant Physician-in-Chief over Physician Education and Development, at Kaiser, Oakland

One of the most important aspects of longitudinal integrated clerkships is the continuity of care provided to patients. This requires that students work after their clinical sessions, with their preceptors, to communicate about patient care. Discussing how to keep track and accomplish such tasks can be a helpful learning experience for students, but it requires clear communication to avoid errors of omission and to encourage student ownership. Let's consider how to communicate clearly with the student when you no longer share a physical space, as there are many opportunities to create teaching moments asynchronously.

Let's first look at students following up on results after a clinical encounter. Lab results can be tricky because first, the student must know how to interpret the result and second, how to communicate the result to their preceptor and possibly to the patient. Some students may have EMR access and preceptors can forward results within this system with a posted question such as "what would you do with this potassium?" or "how would you interpret this urine culture?" Students can be given the opportunity to make a commitment and then can be directed to do further reading on subjects that require more in depth study. If students cannot access the EMR, they can still be assigned the task of following the result, interpreting it, and reaching out to their preceptor. Strategies may include emailing results with identifiers deleted, or even calling students with results. More advanced students may reach out to the patient and provide education and a follow up plan. Similarly, students can follow up on imaging studies ordered for patients. For example, for a chest xray ordered for a patient with a chronic cough, the student can be told "If the xray is normal please call the patient and let her know the results, see how she feels on the medication we prescribed, and forward me a copy of your telephone documentation for me to co-sign. If the xray is abnormal please call or electronically message me before you contact the patient, so we can come up with a plan together." Ideally students can also review primary radiographic studies with radiologists, which is another great learning opportunity.

Physicians are inundated with communications from other providers. These may include specialty consultant reports, PT/OT/Speech therapy reports, or pre-authorizations for medication in some medical systems. Physicians likely also receive communication from patients themselves. There can be value in forwarding copies of reports related to their

patient's care to the student to review between clinic days. Students can learn a tremendous amount from reviewing the care of other interdisciplinary members of the health care team. These reviews shed light on how systems of care affect patients. Students can also be cc'd on responses to patient messages, to virtually "shadow" their doctor's communication within the health record. Keeping tabs of when to share information with the student between visits can prove time consuming and requires mindfulness to the task, but the value gained can be tremendous.

Switching gears a bit, let's talk about how the student can learn about their patient by following the patient through encounters with other providers. Students should have between clinic interactions with our patients, physician consultants, and multidisciplinary team members such as pharmacists and physical therapists. Other podcasts have outlined strategies for encouraging this continuity. Once assigned these types of tasks, students are responsible for sharing with preceptors how the between clinic encounter went, what the next steps were, and what questions arose from joining the clinical encounter.

Lastly, let's discuss common pitfalls that occur when teaching outside of clinic time and assigning student follow up tasks after clinical visits. According to Dr. Ziv, it is important to stress to the student that the preceptor is ultimately responsible, and that the preceptor should be notified immediately around unclear patient questions or comments, or around new medical information that might potentially need action right away. This will help to avoid errors and delay of care. Not knowing is ALWAYS better than guessing and this cannot be overemphasized.

Consistently communicating a time frame or deadline on tasks assigned for follow up between clinic sessions is also important. Understanding the timeline of urgency around checking and communicating results helps the student develop their clinical knowledge, and provides the student with an expectation for safe practice. Consider, for example, is it ok to check the potassium in 3 months after initiating high dose lasix? Well, not really. How about checking the bone density result? Less urgent. That can certainly wait till next week. Although these seem like obvious facts for an experienced preceptor, it may not be something that students have had exposure to and can help broaden clinical knowledge.

Your student should know exactly what you expect between encounters and should be clear about their specific responsibility. Make sure the student realizes that the work done between clinics is also part of their clerkship responsibility and contributes to their overall evaluation. Mindful communication between clinical encounters will allow the preceptor to appropriately and safely delegate tasks. A thoughtful and organized process of communication will also enhance the patient's care experience, both in the areas of quality of care and service experience. Although this takes some effort on the part of the preceptor and the student, the learning gained from between clinic teaching can be invaluable.

Choosing the Right Patients for Continuity

Written by Dr. Joshua Bernstein, Director of the Internal Medicine Clerkship at the Longitudinal Integrated Clerkship at the University of North Carolina School of Medicine, Asheville Campus.

Welcome to another LIC podcast. . Today's topic will be how to choose the best patients for students to see in our continuity clinics. Having students in longitudinal integrated clerkships gives them unique opportunities to learn through patients' experiences as they move through the health care system. Students can follow their patients if admitted, in the emergency room, at a specialist's office, or at ancillary services such as physical therapy. Each encounter provides invaluable learning opportunities in terms of evaluation and management of medical problems and provides students with insight into how patient's medical problems affect patients on a personal level.

As Dr. Bernstein explains, ideally every patient a student sees would be willing and excited to engage with that student. As well, patients should have appropriate medical problems to fill gaps in student knowledge. The patient would be tolerant of the extra time they spend talking to the student before seeing the doctor

What are some strategies for choosing appropriate patients from your practice for students to see in a longitudinal program? Some preceptors just see who is on the schedule for that day and pick out the better patients for students to see or just to have them see whoever is there. However, this leaves a lot to chance and there may be times when the schedule is filled with less than ideal student patients. So, how do we ensure quality patients for our students?

One possible strategy is to start looking for student patients well before the year begins. As patients are seen in the months leading up to the academic year they can be screened and even asked about willingness to work with students. If they are agreeable then instead of saying "I want to see you in 3 months" you would say "I want to see you in 3 months on a Thursday morning, when the students are here. Please tell our assistant when you are checking out that you will be willing to work with a student." You can remind your staff to book these patients in during times when students are present. We find it useful to keep a list of the dates the student is with you tacked to the office wall or on your phone so you can easily make that suggestion. You can also keep a list of possible patients so if there is a day without an appropriate patient for the student scheduled, then your staff could turn to this list to recruit patients. As the year goes by, more and more patients can be added to the list. In Pediatrics this may be the family of 5 kids who seem to come in a lot. In Internal Medicine it may be a patient with multiple medical problems and 2 admissions this year for COPD. For OB it may be a pregnant woman with 2 other kids who is expecting this year, but has Diabetes or Hypertension and may be seen more often for prenatal care. Also consider patients who may need multiple specialists for one issue can also be useful (liver cancer or ESLD, breast cancer, etc).

Another strategy is to keep open slots in the schedule on the day the students are there. A week before, the schedule can be screened and appropriate patients can be identified and moved into student slots. As the year goes on preceptors can meet with their students and try to ascertain if there are any knowledge gaps that need to be filled. A student may not have had exposure to chronic kidney disease for example. The next time you see a patient with this disease then they could be asked about willingness to see a student and if so they could be booked in the next slot when the student is there.

According to Dr. Bernstein, hospital admissions are another unique opportunity to find continuity patients for students. These patients often have multiple medical problems and may be good learning cases for the students. Upon discharge, these patients can be asked to follow up on the student mornings. By reviewing the hospital chart students can get some added hospital medicine knowledge, have some experience in dealing with transitions, of care, and form a relationship with a patient who may have another hospital admission in the future. This works well if you admit your own patients, or you can have the student work with the admitting hospitalist and serve as a link between your office and the hospital team. Students can also be sent to visit a new patient in the hospital, "on behalf" of their primary care doctor.

Another source of patients for the students would be new patients coming into the practice. New patients are an excellent learning opportunity as they come with a blank slate. It is nice if students can have some opportunity to see patients who are not already established. If your practice is closed to new patients then you may consider accepting new patients with the caveat that the patient would be willing to see a student first and would be willing to come on the student day. This has been a very successful technique and has worked well in our clinic.

Lastly, students may bring in new patients that they have had contact with. It is encouraged at the beginning of the year that students invite patients they have had contact with in to our practice, with approval. These patients may come from the hospital wards during inpatient blocks, from ER experiences, from OB rotations, etc. If systems can be established to assure these patients are appropriate for your practice, then this can be an excellent opportunity to get new patients into your office. Once a patient is actively working with students we encourage them to remain in the teaching program and transition to work with subsequent students when their student has moved on to 4th year. This creates continuity for the patients and the students.

This will conclude this podcast on choosing patients for continuity.
