# Supplementary material for: Texting Brief Podcasts to Deliver Faculty Development to Community-Based Preceptors in Longitudinal Integrated Clerkships
Source: MedEdPORTAL. 2018 Sep 21;14:10755. doi: 10.15766/mep_2374-8265.10755 (PMC6342434; doi:10.15766/mep_2374-8265.10755)
Supplement: Supplementary file 1 — A. Podcast 1 Encouraging Continuity.mp3 B. Podcast 2 Bedside Teaching.mp3 C. Podcast 3 Encouraging Student Ownership of Patients.mp3 D. Podcast 4 Communicating and Managing Patient Results During Off-Clinic Days.mp3 E. Podcast 5 Choosing the Right Patients for Continuity.mp3 F. Podcasts 1-5.pdf G. Pre- and Postexperience Surveys.pdf [file mep-14-10755-s001.zip › G._Pre-_and_Postexperience_Surveys.pdf]

Morning Podcast Questionnaire (Pre-Participation)

The following questions are intended to describe your current teaching involvement and strategies for working with your LIC students. We would like to know if brief educational podcasts directed towards LIC preceptors are a useful and feasible means of faculty development.

Thank you for taking the time to complete the below survey.

The below code will be used to keep your assessments together with our post study survey without revealing your identify.

Enter the first letter of your mother's first name ______
Enter the first letter of the month you were born _______
Enter the first letter of the town where you were born ______
Enter the first letter of your middle name ______

What institution are you affiliated with?

FAU MAHEC UCSF/Kaiser

What is your specialty? _______

How many clerkship students do you precept per year? _______

How many months is each student with you per year? _______

How many ½ days/week is each student with you? _____

≤1 1-2 3-4 4-5 5+

How often do you participate in faculty development for improving teaching skills?

Never rarely occasionally often very often

How often do you listen to podcasts for CME credit or medical education?

Never rarely occasionally often very often

How long is your typical commute to work in minutes?

0-10 11-20 21-30 30-40 > 40

Would you listen to a brief faculty development podcast during the time of commute?

Yes Unsure No

How often do you specifically select patients ahead of time who may be good continuity patients for students?

Never rarely occasionally often very often

How often do you communicate with students between clinic sessions?

Never rarely occasionally often very often

How often do you have students present in front of the patient?

Never rarely occasionally often very often

How often do your students follow patients in other health care settings?

Never rarely occasionally often very often

How often do your students contact continuity patients during the week?

Never rarely occasionally often very often

Morning Podcast Questionnaire (Post-Participation)

Thank you for completing the below questionnaire.

This is a follow-up questionnaire to the original pre-questionnaire and aims to see if the faculty development podcasts were useful as an educational tool. It also aims to see if the podcasts had any affect in changing your practice of teaching.

The below code will be used to keep your assessments together with our post study survey without revealing your identify.

Enter the first letter of your mother's first name ______
Enter the first letter of the month you were born _______
Enter the first letter of the town where you were born ______
Enter the first letter of your middle name ______

How many podcasts did you listen to in entirety?

1 2 3 4 5

How helpful did you find the podcasts in improving your clinical teaching skills?

Not helpful at all slightly helpful moderately helpful very helpful

Did the podcasts cause you to change your teaching style?

No Yes

How likely are you to listen to further podcasts?

Not likely at all might likely highly likely

How likely would you be to recommend these podcasts to other colleagues?

Not likely at all might likely highly likely

How often do you specifically select patients ahead of time who may be good continuity patients for students?

Never rarely occasionally often very often

How often do you communicate with students between clinic sessions?

Never rarely occasionally often very often

How often do you have students present in front of the patient?

Never rarely occasionally often very often

How often do your students follow patients in other health care settings?

Never rarely occasionally often very often

How often do your students contact continuity patients during the week?

Never rarely occasionally often very often

Do you have any feedback about the podcasts or suggestions for future expansion of podcasts as a means of faculty development? Please use the space below.

Any Further comments?
