# Supplementary material for: Texting Brief Podcasts to Deliver Faculty Development to Community-Based Preceptors in Longitudinal Integrated Clerkships
Source: MedEdPORTAL. 2018 Sep 21;14:10755. doi: 10.15766/mep_2374-8265.10755 (PMC6342434; doi:10.15766/mep_2374-8265.10755)
Supplement: Supplementary file 1 — A. Podcast 1 Encouraging Continuity.mp3 B. Podcast 2 Bedside Teaching.mp3 C. Podcast 3 Encouraging Student Ownership of Patients.mp3 D. Podcast 4 Communicating and Managing Patient Results During Off-Clinic Days.mp3 E. Podcast 5 Choosing the Right Patients for Continuity.mp3 F. Podcasts 1-5.pdf G. Pre- and Postexperience Surveys.pdf [file mep-14-10755-s001.zip › _Educational_Summary_Report_10755.pdf]

MedEdPORTAL®
The Journal of Teaching and Learning Resources

Original Publication OPEN ACCESS

Texting Brief Podcasts to Deliver Faculty Development to Community-Based Preceptors in Longitudinal Integrated Clerkships

Joshua Bernstein, MD*, Lindsay Mazotti, MD, Tal Ann Ziv, MD, Joanna Drowos, DO, MPH, MBA, Sandra Whitlock, MD, Sarah K. Wood, MD, Shelley L. Galvin, MA, Robyn Latessa, MD
*Corresponding author: bernstein.josh@gmail.com

Citation: Bernstein J, Mazotti L, Ziv TA, et al. Texting brief podcasts to deliver faculty development to community-based preceptors in longitudinal integrated clerkships. *MedEdPORTAL*. 2018;14:10755.
https://doi.org/10.15766/mep_2374-8265.10755

Copyright: © 2018 Bernstein et al. This is an open-access article distributed under the terms of the Creative Commons Attribution license.

Appendices

A. Podcast 1 Encouraging Continuity.mp3
B. Podcast 2 Bedside Teaching.mp3
C. Podcast 3 Encouraging Student Ownership of Patients.mp3
D. Podcast 4 Communicating and Managing Patient Results During Off-Clinic Days.mp3
E. Podcast 5 Choosing the Right Patients for Continuity .mp3
F. Podcasts 1-5.pdf
G. Pre- and Postexperience Surveys.pdf

All appendices are peer reviewed as integral parts of the Original Publication.

Abstract

Introduction: Longitudinal integrated clerkships (LICs) are an increasingly popular clerkship model that relies heavily on community-based preceptors. The availability of an engaged and prepared community-based faculty is crucial to the success of these programs. Teachers in these programs are often geographically separate from medical school campuses, are engaged in busy practices, and have limited time to devote to faculty development activities. **Methods:** We created a series of five brief faculty development podcasts directed towards community-based teachers in LICs from three US medical schools. Topics included encouraging continuity, bedside teaching, encouraging student ownership of patients, communicating and managing patient results between clinic days, and choosing the right patients for continuity. The podcasts were sent via a grouped text message just prior to preceptors' morning commute time. Pre- and postsurveys assessed the acceptability and effectiveness of the podcasts. **Results:** Among the 33 postintervention survey responders, 27 (81.8%) listened to at least three podcasts, 21 (63.6%) found them moderately or very helpful, 23 (69.7%) perceived that the podcasts altered their teaching style, 23 (69.7%) would likely or highly likely listen to further podcasts, and 18 (54.5%) would likely or highly likely recommend the podcasts to colleagues. **Discussion:** In a cohort of multispecialty faculty teaching in LICs, educational podcasts were well received and perceived by preceptors to impact their teaching style. Texting these podcasts to other community-based preceptors may offer an effective strategy for providing faculty development to busy clinicians.

Keywords

Faculty Development, Webcasts, Podcasts, Longitudinal Integrated Clerkships (LICs), Community-Based Faculty, Texting, Text Messaging

Educational Objectives

By the end of this activity, clinical preceptors will be able to:

1. Identify strategies to maximize continuity for medical students and support the development of meaningful relationships with patients.
2. Integrate bedside presentations into teaching in order to model humanism and engage patients as teachers.
3. Formulate strategies to allow student ownership of patient care while increasing student responsibility over time.
4. Direct students to follow and address patient results, reports, and clinic activity between scheduled clinic sessions in order to enhance learning.
5. Identify and assign a cohort of appropriate patients for students to follow throughout their longitudinal integrated clerkship experience.

Introduction

As medical schools rely more on community-based faculty to educate students at clinical sites, the demand for available, engaged, and prepared community-based preceptors is increasing. A 2013 survey

of all deans of medical and allied health professional schools found that more than 80% of respondents in each discipline were concerned about the quantity and quality of clinical training sites. Orienting and training faculty were identified as key obstacles to recruiting and retaining community preceptors and community-based teaching sites.[1] The Alliance for Clinical Education, a national collaborative of eight disciplines' clerkship directors, recently focused on the impending crisis in identifying and training quality clinical preceptors and training sites for learners.[2] Barriers to engagement of preceptors for teaching include differences in job structure in the community, administrative barriers, and lack of knowledge of teaching strategies.[3]

Longitudinal integrated clerkships (LICs) are a curricular innovation relying heavily on community-based faculty for clinical education. The number of medical schools implementing LICs is increasing nationally and internationally as this model is recognized as a successful structure for clinical training.[4] LICs can serve to link community-based health systems with larger academic centers.[5] This link creates opportunities for recruitment of new community-based faculty to precept students. There are well-documented differences in student and faculty experience in LICs that indicate a need for novel teaching techniques for LIC preceptors.[6] Despite this, there are few published resources specifically for faculty teaching in LICs, creating an opportunity to develop innovative faculty development tools.[7,8]

The Liaison Committee on Medical Education recognizes the importance of continued education for teachers and requires medical schools to offer faculty development for community-based preceptors. Physicians themselves value education, as demonstrated in a study comparing physicians who declined teaching opportunities to physicians willing to teach medical students; the latter group considered continuing medical education as one of the primary extrinsic benefits of teaching.[9] However, prior studies have demonstrated the challenges of providing community-based faculty with accessible faculty development. In a recent survey of clerkship directors, preceptor time availability was rated as the most difficult barrier to overcome in delivering faculty development for community-based faculty.[10] Other identified barriers include distance and spread from the medical school campus, diverse training needs, and accessing topics specific to faculty role.[11] In general, faculty development is positively received by community-based faculty, but the optimal format and content of faculty development remain uncertain.[12]

Programs can deliver faculty development materials to community-based teachers through varied approaches. For example, the Preceptor Development Program created by the Mountain Area Health Education Center in Asheville, North Carolina, was able to reach over 500 community preceptors of health professions and medical students. The program distributed materials in formats that included seminars, monographs, web modules, and one-page summary thumbnails.[11] Additional formats described in the literature include a preceptors' LISTSERV, an electronic clinical teaching discussion group, interactive web-based teaching scenarios and cases, CD-ROMs, on-site technology support, and audio content (cassette tapes, CDs, or MP3s).[13,14] Despite the potential flexibility and acceptability of social media for faculty development, few programs have formally embraced this approach.[15] Creative methods to engage community-based faculty in critical faculty development should include newer technologies that are time-saving and available remotely.[10]

Podcasts offer potential as an emerging tool for medical education of students, residents, and fellows and are increasingly popular for educational purposes.[16-18] *MedEdPORTAL* currently cites 34 learning modules using podcasts primarily to educate students and residents on clinical topics. Our podcasts are unique in that topics focus on tools for medical education, with our target audience being faculty preceptors as opposed to traditional learners as listed above. Medical educators can share these podcasts with clinical preceptors specifically to improve their teaching. For resident physicians, podcasts are a preferred method for obtaining medical knowledge.[19] Podcasts created for the education of health care professionals demonstrate popular use nationally and internationally.[20] Existing evidence suggests podcasts are well

received and are an effective pedagogic means of improving learning outcomes.[21-23] Despite the potential of podcasts as an educational tool, little research has evaluated their use for faculty development of community-based preceptors. The effectiveness and acceptability of podcasts for faculty development, followed by virtual workshops, were verified in one small study in a cohort (13) of family medicine preceptors teaching in a longitudinal program.[24] Larger scale efforts examining the use of podcasts exclusively for faculty development across preceptor medical specialties have not yet been reported in the literature.

We developed brief faculty development podcasts, disseminated them to community-based faculty via text message, and tracked users' engagement and satisfaction with this approach. The use of cell phones to text brief podcasts just prior to morning commute time was hypothesized to overcome identified barriers, such as time and geographic distance. The project also aimed to satisfy community-based preceptors' desire for continued medical education and to improve their teaching practices.

Methods

Participants/Target Audience

We invited all outpatient community-based preceptors teaching in LICs from the University of North Carolina (UNC) School of Medicine Asheville campus, the Charles E. Schmidt College of Medicine at Florida Atlantic University (FAU), and the University of California San Francisco–Kaiser Permanente (UCSF-KP) to participate. Faculty could be from the specialties of family medicine, general surgery, internal medicine, obstetrics and gynecology, pediatrics, psychiatry, or neurology.

Topic Selection

A working group of faculty leadership from the above three institutions met and, through review of the literature and discussion, generated a list of 21 faculty development topics focusing on community-based preceptors teaching in LICs. We selected five topics felt to be of highest importance to develop into podcasts. The selected topics are described below, with specific learning objectives outlined.

Encouraging Continuity (Appendix A)

This podcast focuses on methods preceptors can use to encourage students to follow their patients over time and into different health care settings outside of the clinic. The learning objectives for this podcast are as follows:

- Appreciate the opportunity to develop meaningful relationships with patients through continuity over time in LICs.
- Identify opportunities for continuity during office visits and within the office-based practice for LIC students.
- Direct student involvement with patients in other health care settings or during hospital admissions within LICs.
- Reinforce strategies that promote continuous student engagement with patients in the office, in the hospital, and across health care settings.

Bedside Teaching (Appendix B)

This podcast provides an evidence-based review, encouraging faculty to have students present the history and clinical findings to preceptors in front of the patient in the exam room. The learning objectives for this podcast are as follows:

- Realize challenges that prevent students from delivering patient presentations at the bedside, in front of patients.

- Define patient engagement as a valued contribution to patients' own care and to the educational process through bedside teaching.
- Describe benefits of bedside teaching such as increased efficiency and patient satisfaction through engaging patients in student education.
- Demonstrate patient-centered, humanistic care through bedside teaching with students in LICs.

Encouraging Student Ownership of Patients (Appendix C)

This podcast explores how preceptors can safely allow students progressive responsibility and independence in the care of patients over time. The learning objectives for this podcast are as follows:

- Describe student ownership as a progressive responsibility and key component of caring for patients in LICs.
- Employ the FACT mnemonic (follow-through, autonomy/advocacy, communication/commitment, and teamwork) to foster student ownership of patients in LICs.
- Realize the importance of the office environment and role modeling in encouraging student ownership of patients in LICs.
- Support student ownership and engagement through intentionally identifying a smaller cohort of patients within the practice for an LIC student to closely follow.

Communicating and Managing Patient Results During Off-Clinic Days (Appendix D)

This podcast shares examples of how preceptors can teach and have students remain engaged with their patients between clinic sessions through sharing and teaching about patient results and clinic activity. The learning objectives for this podcast are as follows:

- Advance student learning by supporting patient continuity and follow-up following clinical visits in LICs.
- Create strategies to organize, track, and resolve patient-related results for student learning and engagement following LIC clinic visits.
- Arrange opportunities for students to review communications pertaining to patients for learning and engagement opportunities between clinic visits in LICs.
- Reinforce the importance of students understanding the responsibility of communicating patient information or any uncertainty with preceptors to ensure safe patient care in LICs.

Choosing the Right Patients for Continuity (Appendix E)

This podcast discusses techniques to intentionally select the most appropriate patients for students to see in order to maximize the experience for the learner and the patient through promoting continuity of relationships. The learning objectives for this podcast are as follows:

- Identify willing and medically appropriate patients for LIC students to follow in their clerkships.
- List strategies for identifying appropriate patients for LIC students to care for during clinic sessions.
- Engage LIC students in caring for hospitalized patients or patients new to the practice as a strategy for building relationships and continuity with students.
- Communicate the importance of LIC students scheduling follow-up with patients they have encountered in other settings with their outpatient/LIC preceptor to promote continuity and meaningful relationships in clerkships.

Podcast Development and Distribution

The podcast scripts (Appendix F) were created and written with the goal of keeping the listening length between 5 and 7 minutes. The final podcast segments were peer edited and reviewed by coauthors. They

were then recorded to MP3 format. An Excel spreadsheet with participating faculty cell phone numbers was created, divided by time zones, and downloaded to an Apple iPhone. This was used to cut and paste 20 phone numbers at a time (the maximum allowed) into the messaging app to be texted in a group text. The series of five brief podcasts was texted to community-based preceptors teaching in LICs at the three medical schools every other week over a 10-week period. They were specifically sent at 7:30 a.m. (either Eastern or Pacific time) with the goal of having preceptors receive them while preparing for a morning commute.

Evaluation Methodology
Preceptors were initially invited via group email and were given the option to receive the podcasts, to receive the podcasts and participate in data collection, or to decline to participate in both the faculty development podcasts and accompanying data collection. The initial email outlined the purpose of the podcasts and the project. Two weeks later, an affiliated medical school contact sent a follow-up invitation to nonresponders. We sent a preparticipation survey link to participating preceptors using an online survey tool (SurveyMonkey) 2 weeks prior to the initiation of the podcasts. Following the podcast distribution over 10 weeks, we emailed a postparticipation survey link through SurveyMonkey (Appendix G). Nonresponding preceptors were emailed a follow-up reminder and survey link. Participants were asked to create a four-letter identification code to link the anonymous survey responses from both before and after podcast exposure. Community-based preceptor satisfaction and interest in receiving additional podcasts were measured. This study received exemptions from the Institutional Review Boards at Mission Health (UNC Asheville), FAU, and UCSF-KP.

Results

Of 235 outpatient community-based preceptors invited to participate, 88 agreed to both receive the podcasts and participate in surveys, with a response rate of 37.4%. Of the original 88 participants, 68 (77.3%) completed the preintervention survey, and 33 (37.5%) completed the postintervention survey. Prior to the intervention, 67 respondents reported university affiliation, which was well distributed: 15 (22.4%) at UCSF-KP, 25 (37.3%) at UNC Asheville, and 27 (40.2%) at FAU. Preceptor specialties represented among those completing the survey spanned all the disciplines, including family medicine, general surgery, internal medicine, obstetrics and gynecology, pediatrics, psychiatry, and neurology. Of the 67 faculty responding, 43 preceptors attested to never (15, 22.4%) or rarely (28, 41.8%) receiving faculty development.

Among the 33 postintervention survey responders, 12 (36.4%) listened to all five podcasts, five (15.2%) listened to four podcasts, and 10 (30.3%) listened to three podcasts, with a total of 27 (81.8%) having listened to at least three podcasts. Of respondents, 21 (63.6%) found the podcasts moderately or very helpful, 23 (69.7%) perceived that the podcasts altered their teaching style, 23 (69.7%) were likely or highly likely to listen to further podcasts, and 18 (54.5%) were likely or highly likely to recommend the podcasts to colleagues.

Twenty-three participants completed both pre- and postsurveys and listened to a median of four podcasts (range: one to five). While the percentage of preceptors reporting often or very often employing each specific educational strategy was overall greater among the postintervention respondents as compared to the preintervention respondents, changes in reported frequency of use (never to very often) among fully participating participants who completed both surveys (pre- vs. postintervention) did not reach statistical significance (see the Figure; *p*s = 1.000, .906, .730, .635, and .361, respectively). There was, however, a 36.4% increase in the percentage of preceptors reporting often or very often having students present history and physical exam findings in front of their patients (Figure).

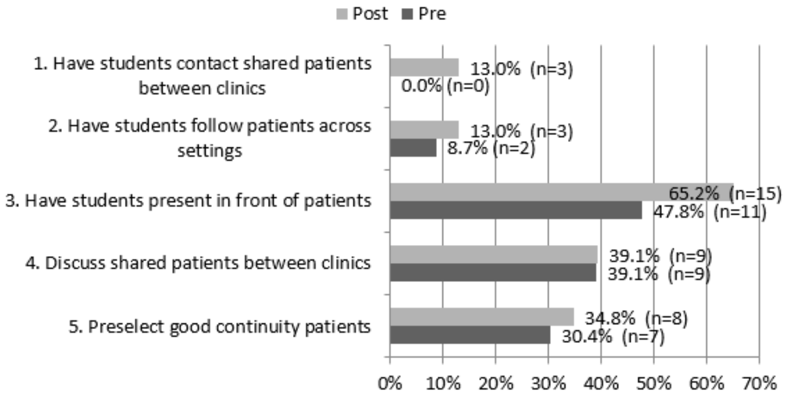

Figure. Percentage of fully participating preceptors reporting engaging in educational behaviors with medical students often to very often (*N* = 23). Post, postintervention; pre, preintervention.

Discussion

The increasing use of community-based educators for medical school training, as well as the expansion of innovative curricular structures such as LICs, has intensified the need for timely and effective faculty development. We demonstrated that educational podcasts, texted to community-based preceptors, are well received and are a promising pedagogic tool for faculty teaching medical students in LICs.

We created short, high-yield content specific to skills faculty need for LIC teaching. Podcasts in medical education vary in length, and the ideal duration for these has yet to be determined.[25] Given the limited time community-based faculty have available for educational activities, we hypothesized that a short duration would be better received by and more accessible to busy clinicians. Most preceptors listened to at least three podcasts, but only one-quarter of participants listened to all five podcasts. Preceptor time availability is a known barrier to faculty development.[10] We felt this was one of the most likely explanations for the relatively small number of preceptors completing all five podcasts. Other barriers may have been lack of perceived value by preceptors, receiving the podcasts via text at an inconvenient time, or preference by certain preceptors or groups of faculty for a different type of learning modality. Future projects could explore optimization of podcast length, comparing brief reviews to longer, more in-depth topics, and could specifically address other barriers to participation.

We did not collect data on which specific podcasts preceptors chose to listen to. It is possible that certain topics seemed more or less relevant to specific preceptors or specialty groups. Podcast titles could have played a role in preceptors' sense of perceived value. Initial faculty preparticipation surveys were well dispersed by specialty, but we did not evaluate whether certain specialties were more likely to listen to podcasts than others. Future studies could evaluate whether trends exist that could allow tailoring of specific podcasts towards specific faculty groups.

Texting was used as the primary mechanism for disseminating the podcasts to faculty. We hypothesized that directly delivering podcasts to preceptors would increase frequency of use. There is evidence that using text messaging as a mechanism of dissemination of medical knowledge can be effective at a graduate level.[26] However, many faculty fall not in the millennial generation but in either Generation X or the Baby Boomer generation, making comparisons between these groups difficult. Future projects could compare the effectiveness and desirability of text delivery versus more traditional methods of knowledge acquisition, such as websites, podcast channels, or email, and whether age or generation contributes to desired delivery method.

We chose to text the podcast during morning commute times with the hypothesis that faculty may have a brief window of undisturbed time to listen to the podcast while commuting to work, thus increasing

podcast usage. Future studies could ascertain whether there is an ideal time for preceptors to receive podcasts. The podcasts were texted using a standard smartphone messaging app requiring batching of numbers into groups of 20 preceptors at a time and also requiring separate time-zone groups to be messaged later in the day. Technology exists to allow larger group texts, and other programs may choose to invest in this technology to increase efficiency of delivery.

We were able to ascertain the acceptability of these podcasts as a faculty development tool. The majority of responding preceptors felt the podcasts changed their teaching behavior. Trends toward changes in teaching style were seen without statistical significance, particularly in having students present history and physical examination findings in front of patients. We were limited by a low survey response rate, which decreased power to detect significant change in teaching style. The podcasts were sent during the summer of 2016, when many community-based faculty may have been on vacation. This may have negatively impacted podcast participation rates. Projects scheduled at other times of the year, particularly at the beginning of a new LIC year, may yield higher participation. More direct methods of contacting busy preceptors who do not respond may be effective at increasing participation. Rewarding preceptors for participation, including offering continuing medical education credit, could be used as a means to increase participation rates.

In addition, we were limited by the self-report nature of the data collected. This limitation precluded us from fully evaluating preceptors' achievement of the learning objectives. Future implementations of the podcasts could solicit or compare students' feedback on preceptor teaching performances before and after the intervention.

Clear consensus exists regarding the need to recruit and retain high-quality community-based preceptors who are prepared to teach medical students. Quality professional development for these educators is crucial. However, there are many known barriers to successful delivery of content.[27] We have proposed a solution for an area of needed faculty development by focusing on core strategies used in LIC teaching. We conclude that these five podcasts, delivered via text message just prior to morning commute time, provide a viable and important option for faculty development for community-based preceptors. In an era of multitasking, commuting, and 24-hour connectedness, educational leaders may find that brief, just-in-time, accessible content is preferred in order to reach busy clinicians and distributed community-based faculty.

Joshua Bernstein, MD: Clinical Assistant Professor, Internal Medicine, UNC Health Sciences at MAHEC; Internal Medicine Course Director, Education and Development, University of North Carolina School of Medicine, Asheville campus

Lindsay Mazotti, MD: Associate Professor, Clinical Medicine, Kaiser Permanente East Bay—University of California, San Francisco, School of Medicine; Assistant Physician in Chief, Education and Development, Kaiser Permanente East Bay—University of California, San Francisco, School of Medicine

Tal Ann Ziv, MD: Associate Professor, Clinical Medicine, Kaiser Permanente East Bay—University of California, San Francisco, School of Medicine; Associate Program Director, Longitudinal Integrated Clerkship, Kaiser Permanente East Bay—University of California, San Francisco, School of Medicine

Joanna Drowos, DO, MPH, MBA: Associate Professor, Family Medicine, Charles E. Schmidt College of Medicine at Florida Atlantic University; Associate Chair, Integrated Medical Science Department, Charles E. Schmidt College of Medicine at Florida Atlantic University; Clerkship Director, Community and Preventive Medicine Clerkship, Charles E. Schmidt College of Medicine at Florida Atlantic University

Sandra Whitlock, MD: Assistant Professor, Internal Medicine, UNC Health Sciences at MAHEC; Associate Program Director, University of North Carolina School of Medicine, Asheville campus

Sarah K. Wood, MD: Associate Professor, Pediatrics, Charles E. Schmidt College of Medicine at Florida Atlantic University; Senior Associate Dean, Medical Education, Charles E. Schmidt College of Medicine at Florida Atlantic University

Shelley L. Galvin, MA: Adjunct Assistant Professor, Department of Obstetrics and Gynecology, MAHEC Center for Research, University of North Carolina School of Medicine; Director of Research, Department of Obstetrics and Gynecology, MAHEC Center for Research, University of North Carolina School of Medicine

Robyn Latessa, MD: Professor, Family Medicine, UNC Health Sciences at MAHEC; Director, University of North Carolina School of Medicine, Asheville campus; Assistant Dean, University of North Carolina School of Medicine, Asheville campus

Acknowledgments
The authors are grateful to Samuel Porter, MD, for his assistance in recording the podcasts to MP3 format; Gimol George, EdD, for her help in reviewing surveys; Laurel DeCou, MFA, for her help coordinating survey distribution; and Sue Stigleman, MLS, for her help with resources and literature review.

Disclosures
None to report.

Funding/Support
None to report.

Ethical Approval
The Institutional Review Boards at Mission Health (University of North Carolina–Asheville), Florida Atlantic University, and University of California San Francisco–Kaiser Permanente approved this study.

References

1. *Recruiting and Maintaining U.S. Clinical Training Sites: Joint Report of the 2013 Multi-Discipline Clerkship/Clinical Training Site Survey*. Washington, DC: Association of American Medical Colleges; 2014.
2. Christner JG, Dallaghan GB, Briscoe G, et al. The community preceptor crisis: recruiting and retaining community-based faculty to teach medical students—a shared perspective from the Alliance for Clinical Education. *Teach Learn Med.* 2016;28(3):329-336. https://doi.org/10.1080/10401334.2016.1152899
3. Piggott T, Morris C, Lee-Poy M. Preceptor engagement in distributed medical school campuses. *Can Med Educ J*. 2015;6(2):e20-e28.
4. Worley P, Couper I, Strasser R, et al; for Consortium of Longitudinal Integrated Clerkships (CLIC) Research Collaborative. A typology of longitudinal integrated clerkships. *Med Educ*. 2016;50(9):922-932. https://doi.org/10.1111/medu.13084
5. Poncelet AN, Mazotti LA, Blumberg B, Wamsley MA, Grennan T, Shore WB. Creating a longitudinal integrated clerkship with mutual benefits for an academic medical center and a community health system. *Perm J*. 2014;18(2):50-56. https://doi.org/10.7812/TPP/13-137
6. Teherani A, O'Brien BC, Masters DE, Poncelet AN, Robertson PA, Hauer KE. Burden, responsibility, and reward: preceptor experiences with the continuity of teaching in a longitudinal integrated clerkship. *Acad Med*. 2009;84(10):S50-S53. https://doi.org/10.1097/ACM.0b013e3181b38b01
7. Latessa R, Schmitt A, Beaty N, Buie S, Ray L. Preceptor teaching tips in longitudinal clerkships. *Clin Teach*. 2016;13(3):213-218. https://doi.org/10.1111/tct.12416
8. Levitt DS, Cooke M. Tips for teaching in longitudinal clerkships.*Clin Teach*. 2011;8(2):93-96. https://doi.org/10.1111/j.1743-498X.2010.00420.x
9. Ryan MS, Vanderbilt AA, Lewis TW, Madden MA. Benefits and barriers among volunteer teaching faculty: comparison between those who precept and those who do not in the core pediatrics clerkship. *Med Educ Online*. 2013;18(1):20733. https://doi.org/10.3402/meo.v18i0.20733
10. Drowos J, Baker S, Harrison SL, Minor S, Chessman AW, Baker D. Faculty development for medical school community-based faculty: a Council of Academic Family Medicine Educational Research Alliance study exploring institutional requirements and challenges. *Acad Med*. 2017;92(8)1175-1180. https://doi.org/10.1097/ACM.0000000000001626
11. Langlois JP, Thach SB. Bringing faculty development to community-based preceptors.*Acad Med*. 2003;78(2):150-155. https://doi.org/10.1097/00001888-200302000-00009
12. Bramson R, VanLandingham A, Heads A, Paulman P, Mygdal W. Reaching and teaching preceptors: limited success from a multifaceted faculty development program. *Fam Med.* 2007;39(6):386-388.
13. Cook DA, Steinert Y. Online learning for faculty development: a review of the literature. *Med Teach.* 2013;35(11):930-937. https://doi.org/10.3109/0142159X.2013.827328
14. Willett LR. Brief report: utilizing an audiotape for outpatient preceptor faculty development.*J Gen Intern Med.* 2006;21(5):503-505. https://doi.org/10.1111/j.1525-1497.2006.00437.x
15. Cahn PS, Benjamin EJ, Shanahan CW. "Uncrunching" time: medical schools' use of social media for faculty development.*Med Educ Online*. 2013;18(1):20995. https://doi.org/10.3402/meo.v18i0.20995

16. Cadogan M, Thoma B, Chan TM, Lin M. Free open access meducation (FOAM): the rise of emergency medicine and critical care blogs and podcasts (2002–2013). *Emerg Med J.* 2014;31(e1):e76-e77. https://doi.org/10.1136/emermed-2013-203502
17. Matava CT, Rosen D, Siu E, Bould DM. eLearning among Canadian anesthesia residents: a survey of podcast use and content needs. *BMC Med Educ.* 2013;13:59. https://doi.org/10.1186/1472-6920-13-59
18. Lin M, Thoma B, Trueger NS, Ankel F, Sherbino J, Chan T. Quality indicators for blogs and podcasts used in medical education: modified Delphi consensus recommendations by an international cohort of health professions educators. *Postgrad Med J.* 2015;91(1080):546-550. https://doi.org/10.1136/postgradmedj-2014-133230
19. Mallin M, Schlein S, Doctor S, Stroud S, Dawson M, Fix M. A survey of the current utilization of asynchronous education among emergency medicine residents in the United States. *Acad Med.* 2014;89(4):598-601. https://doi.org/10.1097/ACM.0000000000000170
20. Nwosu AC, Monnery D, Reid VL, Chapman L. Use of podcast technology to facilitate education, communication and dissemination in palliative care: the development of the AmiPal podcast. *BMJ Support Palliat Care.* 2017;7(2):212-217. https://doi.org/10.1136/bmjspcare-2016-001140
21. Edmond M, Neville F, Khalil HS. A comparison of teaching three common ear, nose, and throat conditions to medical students through video podcasts and written handouts: a pilot study. *Adv Med Educ Pract.* 2016;7:281-286. https://doi.org/10.2147/AMEP.S101099
22. Vasilopoulos T, Chau DF, Bensalem-Owen M, Cibula JE, Fahy BG. Prior podcast experience moderates improvement in electroencephalography evaluation after educational podcast module. *Anesth Analg.* 2015;121(3):791-797. https://doi.org/10.1213/ANE.0000000000000681
23. Raupach T, Grefe C, Brown J, Meyer K, Schuelper N, Anders S. Moving knowledge acquisition from the lecture hall to the student home: a prospective intervention study. *J Med Internet Res.* 2015;17(9):e223. https://doi.org/10.2196/jmir.3814
24. Delver H, Jackson W, Lee S, Palacios M. FM POD: An evidence-based blended teaching skills program for rural preceptors*Fam Med.* 2014;46(5):369-377.
25. Singh D, Alam F, Matava C. A critical analysis of anesthesiology podcasts: identifying determinants of success. *JMIR Med Educ.* 2016;2(2):e14. https://doi.org/10.2196/mededu.5950
26. Mount HR, Zakrajsek T, Huffman M, et al. Text messaging to improve resident knowledge: a randomized controlled trial. *Fam Med.* 2015;47(1):37-42.
27. Searle NS, Thibault GE, Greenberg SB. Faculty development for medical educators: current barriers and future directions.*Acad Med.* 2011;86(4):405-406. https://doi.org/10.1097/ACM.0b013e31820dc1b3

Received: February 26, 2018 | **Accepted:** August 22, 2018 | **Published:** September 21, 2018
